# *DIA1R* Is an X-Linked Gene Related to *Deleted In Autism-1*

**DOI:** 10.1371/journal.pone.0014534

**Published:** 2011-01-17

**Authors:** Azhari Aziz, Sean P. Harrop, Naomi E. Bishop

**Affiliations:** Department of Microbiology, La Trobe University, Bundoora, Victoria, Australia; Deutsches Krebsforschungszentrum, Germany

## Abstract

**Background:**

Autism spectrum disorders (ASDs) are frequently occurring disorders diagnosed by deficits in three core functional areas: social skills, communication, and behaviours and/or interests. Mental retardation frequently accompanies the most severe forms of ASDs, while overall ASDs are more commonly diagnosed in males. Most ASDs have a genetic origin and one gene recently implicated in the etiology of autism is the *Deleted-In-Autism-1* (*DIA1*) gene.

**Methodology/Principal Findings:**

Using a bioinformatics-based approach, we have identified a human gene closely related to *DIA1*, we term *DIA1R (DIA1-Related)*. While *DIA1* is autosomal (chromosome 3, position 3q24), *DIA1R* localizes to the X chromosome at position Xp11.3 and is known to escape X-inactivation. The gene products are of similar size, with *DIA1* encoding 430, and *DIA1R* 433, residues. At the amino acid level, DIA1 and DIA1R are 62% similar overall (28% identical), and both encode signal peptides for targeting to the secretory pathway. Both genes are ubiquitously expressed, including in fetal and adult brain tissue.

**Conclusions/Significance:**

Examination of published literature revealed point mutations in *DIA1R* are associated with X-linked mental retardation (XLMR) and *DIA1R* deletion is associated with syndromes with ASD-like traits and/or XLMR. Together, these results support a model where the *DIA1* and *DIA1R* gene products regulate molecular traffic through the cellular secretory pathway or affect the function of secreted factors, and functional deficits cause disorders with ASD-like symptoms and/or mental retardation.

## Introduction

Autism spectrum disorders (ASDs) encompass a variety of syndromes, diagnosed on the basis of three core symptoms: deficits in social skills, impaired use of language, and restricted and stereotypic behaviours and/or interests [1]. Classical ‘autistic disorder’ (AD) falls under the ASD umbrella and, in addition to the three core symptoms, is frequently accompanied by mental retardation, as indicated by an intelligence quotient (IQ) of <70 [2]–[11]. By contrast, presence of the core ASD symptoms accompanied by an average (or above) IQ, is typically classified as Asperger syndrome or high-functioning autism [6], [12], [13]. The term pervasive development disorder (PDD) is often used as a broader diagnostic descriptor, to encompass not only all ASDs, but also PDD-not-otherwise-specified (PDD-NOS or atypical autism), Rett disorder and childhood disintegrative disorder [14]. ASDs cause morbidity in as many as 1∶60 people, with the incidence being as high as 1∶40 in males [15]–[19].

ASDs have an almost exclusive genetic origin [20]–[25]. The concordance rate for monozygotic twins, from various studies, ranges from 36% to as high as 100% in one study on female twins, while concordance rates of dizygotic twins are reported to vary from 0% to 31%, with values as high as 40% for male-male twin pairs, and heritability estimates of ∼90% have been calculated [21], [25]–[35]. In twin studies, when a broader definition of ASD is used, higher concordance rates are found; while lower rates are determined when a stricter definition of autistic disorder is used. These findings indicate that the severity of ASD symptoms (e.g. AD versus AS/HFA or PDD-NOS) and the age of diagnosis can differ between monozygotic twins diagnosed with ASD [26], [28]–[30], [34]. As a result, epigenetic influences have been suggested to contribute to the overall ASD phenotype [36]–[39]. Recent data also indicate a further ∼10% of ASD cases are caused by spontaneous genetic mutations [40]–[43]. Currently, more than 25 different loci encode ASD-susceptibility genes, with many more being investigated [44]. Some, but not all, of the genes implicated in ASD overlap with those implicated in MR [45]–[48]. Nonetheless, no genetic susceptibility locus accounts for more than a small percentage of ASD cases [49]. While these findings demonstrate an etiological heterogeneity for ASDs, many of the genes appear to interact at the level of molecular pathways, making it likely that mutations of one ASD-implicated gene may affect the function of others [50]. These findings suggest a common pathway(s) for the etiology of ASDs and raise hope that common treatments may be developed.

It is rare for those with ASDs to lead independent lives in adulthood, and those with severe intellectual disability (IQ<50) have the poorest outcomes [51]–[55]. By contrast, the outcome is improved for those with an ASD who lack accompanying mental retardation (MR), and 10–15% of those with Asperger syndrome/high-functioning ASD achieve independence in adulthood [55]–[57]. While these studies highlight the debilitating life-long effects of ASDs, they emphasize the additional problems conferred by the presence of intellectual disability. As with ASDs, X-linked mental retardation (XLMR) is more common in males and also has a heterogenous genetic etiology [58]. MR affects around 2% of the population and accounts for 5–10% of the health care budget in developed countries [58], with XLMR representing between 5–15% of all MR [59]. Mutations in approximately 10% of the 2,000 genes on the human X chromosome can cause XLMR, although the cellular role of most of these genes remains uncharacterized [60].

We report the identification of a human gene related to the recently identified *Deleted In Autism 1* (*DIA1*) gene. The *DIA1* gene was identified in a genetic study for ASD genes [61], where it was found that hemizygous *DIA1* deletions were asymptomatic, while a homozygous *DIA1* deletion coincided with a classical autism diagnosis. Using a bioinformatics-based approach, we have identified a *DIA1*-related gene, we term *DIA1R*, which localizes to the human X chromosome at position Xp11.3. Deletion and/or mutation of human *DIA1R* is associated with ASD-like syndromes and/or XLMR.

## Results

### Identification of a human *DIA1*-related gene

**Figure 1:**
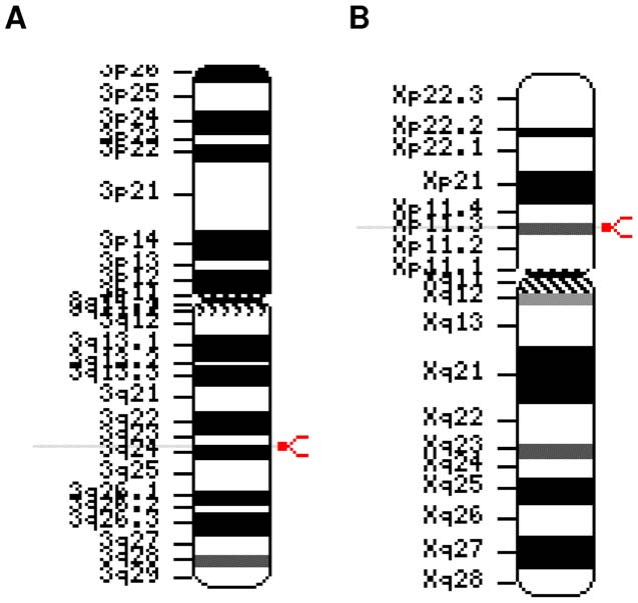
Chromosomal ideogram representation of the location of human *DIA1 (c3orf58)* and *DIA1R (cXorf36)*. (A) *DIA1* localization on human chromosome 3 at position 3q24. (B) *DIA1R* localization on the human X chromosome at position Xp11.3. Gene localization is indicated by a modified red arrowhead on the right-hand side, and a light-grey line extending through the text on the left-hand side.

The human *c3orf58* gene, found at chromosome position 3q24, has recently been renamed *DIA1* on the basis of its deletion in ASD [61]. Human *DIA1* has a known orthologue in both the mouse (*Mus musculus*) and rat (*Rattus norvegicus*) genomes, where it is alternatively called *1190002N15Rik*, *Ab2-095* or *GoPro49* [62]. A search of the HomoloGene database [63], revealed further *DIA1* orthologues in the chimpanzee (*Pan troglodytes*), dog (C*anis familiaris*), cow (*Bos taurus*), mouse (*Mus musculus*), chicken (*Gallus gallus*), and zebrafish (*Danio rerio*) genomes (data not shown; [64]). Unexpectedly, a Basic Local Alignment Search Tool (BLAST) search [65] of the non-redundant (NCBI) database [63], [66], using the sequence of the human *DIA1* gene product, revealed a significantly similar human gene product, LOC79742. This gene product was encoded by the *cXorf36* gene (for accession numbers and identifiers, see Table 1) and localized to the human X chromosome at position Xp11.3 (Figure 1). We have renamed this gene *DIA1R* (*DIA1-Related*). A search of the HomoloGene database [63] revealed orthologues of *DIA1R* in the cow, mouse, chicken, and zebrafish genomes (data not shown).

**Table 1:** Accession numbers and identifiers for human *DIA1* and *DIA1R*.

Database	*DIA1* identifier or accession number	*DIA1R* identifier or accession number
HUGO gene symbol*	c3orf58	cXorf58
RefSeq** protein	NP_775823	NP_789789
RefSeq mRNA	NM_173552	NM_176819
RefSeq chromosome	NC_000003	NC_000023
GenInfo (GI)	27734895	193804854
ENSEMBL protein	ENSP00000320081	ENSP00000381086
ENSEMBL transcript	ENST00000315691	ENST00000398000
ENSEMBL gene	ENSG00000181744	ENSG00000147113
UniProt	Q8NDZ4	Q9H7Y0
UniGene	Hs.288954	Hs.98321
Entrez gene GeneID	205428	79742
HGNC***	28490	25866
Aliases	LOC205428MGC33365GoPro49Ab2-095DIA1	LOC79742DKFZp313K0825EPQL1862FLJ55198FLJ14103PRO3743bA435K1.1FLJ55198

*Human genome organization (HUGO) official gene name.

**NCBI non-redundant reference sequence [63].

***HUGO gene nomenclature committee (HGNC) identifier.

### Comparison of the human *DIA1* and *DIA1R* gene products

**Figure 2:**
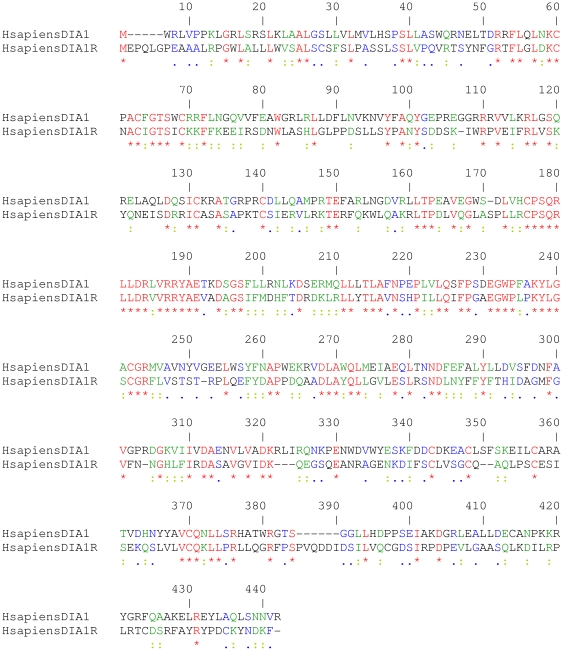
Amino acid sequence comparison of human DIA1 and human DIA1R. The sequence alignment was generated using CLUSTALX [164]. Identical amino acids are highlighted in red font and indicated below the alignment with an asterisk (*). Strongly similar amino acids are highlighted in green font and indicated below the alignment with a colon (:). Weakly similar amino acids are highlighted in blue font and indicated below the alignment with a full stop (.). Dissimilar amino acids are in black font. Amino acid numbering is provided above the alignment. Human DIA1 is 430, and DIA1R 433, amino acids in their immature forms (before signal peptide cleavage). Gaps required for optimal alignment are indicated by dashes. The two proteins have 28% identical, 21% strongly similar, and 13% weakly similar, residues. Standard single-letter amino acid abbreviations are used.

The human *DIA1* and *DIA1R* genes encode gene products of 430 and 433 residues, respectively, with predicted molecular weights of 49.5 and 48.6 kDa. To compare the human DIA1 and DIA1R proteins with each other, we used two methods: BLAST analyses and amino acid alignments. Pair-wise protein BLAST analyses generate ‘expect values’ (E-values), where values less than one are considered significantly similar, and the smaller the E-value, the greater the similarity [65]. E-values of 2e-42 and 1e-42 were obtained for reciprocal BLAST searches, comparing the human *DIA1* gene product with that of *DIA1R*, or vice versa. Next, amino acid alignments were employed to compare the human DIA1 and DIA1R proteins at the amino acid level (Figure 2). Amino acid alignments revealed human DIA1 and DIA1R are 62% similar overall (28% identical, and 34% similar, residues), with similarity greater in the central portion of the two proteins, compared to the amino- or carboxy-terminal regions. This similarity suggests that the central region of *DIA1* and *DIA1R* encodes a biological function common to the two gene products.

### Signal peptides in human DIA1 and DIA1R

Most proteins transported to the endoplasmic reticulum (ER) have a stretch of hydrophobic amino acids at their amino-terminus, known as the signal peptide (SP), which are recognized by the signal recognition particle (SRP) to facilitate ER import [67]. The length of signal peptides usually falls within a range of 5–60 residues. Subsequent to SRP recognition, SPs are typically cleaved from the imported pre-protein by signal peptide peptidase activity [68]. Most imported proteins are then transported from the ER to the Golgi apparatus [69]. As determined above, the most disparate region found when DIA1 and DIA1R are aligned (Figure 2) is the extreme amino-terminus. In a previous assessment of the rat *DIA1* gene product, the presence of a predicted signal peptide was detected, and functionally supported by localization of DIA1 to the lumen of the Golgi apparatus [62]. We therefore analyzed the human *DIA1* and *DIA1R* gene products for the presence of amino-terminal signal peptides.

There are a variety of algorithms designed to predict the presence of signal peptides, and we used three of the best-performing algorithms [70], [71] to both analyze the *DIA1* and *DIA1R* gene products for signal peptides, and to predict the signal peptide cleavage sites. The methodologies used were: (i) the neural network (NN) algorithm of SignalP v3.0 [72], (ii) the hidden Markov model (HMM) of SignalP v3.0 [72], and (iii) the SigCleave algorithm [73] at EMBOSS [74]. No trans-membrane domains were predicted, nor ER-retrieval or retention motifs, in either DIA1 or DIA1R (data not shown).

**Figure 3:**
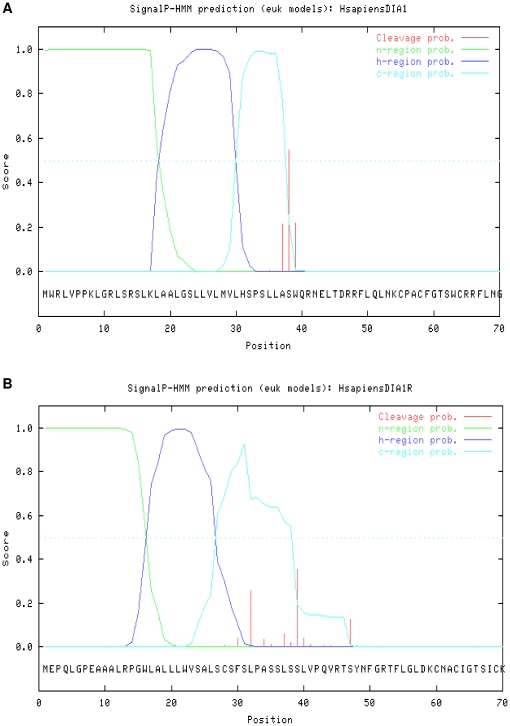
Prediction of signal peptides in human DIA1 and DIA1R. The amino acid sequence of (A) human DIA1 or (B) human DIA1R, was evaluated for amino-terminal signal peptides using the hidden Markov model of SignalP v3.0 [72]. C-region (cleavage-recognition region) scores are in aqua, H-region (hydrophobic region) scores are in dark blue, N-region (N-terminal signal peptide sequence) scores are in green, and the cleavage probability (that a given amino acid is the first in the mature protein) indicated in red. The significance cut-off value for all probability scores is 0.5, and is indicated by a blue dotted line. Standard single-letter amino acid abbreviations and numbering is provided below the graph.

Our analyses revealed SPs in human DIA1 and DIA1R, using all three prediction methods (Figures 3 and S1; data not shown). However, while all methods predicted a SP in both DIA1 and DIA1R, some methods used had varying success in pin-pointing the exact cleavage site used to generate the mature protein. The HMM algorithm of SignalP confidently (score of 0.544, where 0.5 is considered significant) predicted the signal peptide cleavage site of human DIA1 (Figure 3A) as between amino acids 37 and 38 (SLLA-SWQR using single-letter amino-acid code, where the hyphen indicates the site of cleavage). The same site was also the highest-scoring cleavage site predicted by SigCleave (score 7.79, where a score over 3.50 is considered significant), providing confidence that this is the site of human DIA1 SP cleavage. By contrast, the NN algorithm did not predict a DIA1 cleavage site with a significant score (Figure S1A). For human DIA1R, two cleavage sites were suggested by the HMM algorithm (Figure 3B), but neither score passed into the significant range (0.26 for cleavage between amino acids 31 and 32, 0.35 for between amino acids 38 and 39, where 0.5 is considered significant). The NN algorithm (Figure S1B) only predicted a single DIA1R cleavage site (between amino acids 31 and 32) with a Y-score close to significance (a Y-score of 0.44, where a Y-max value of 0.5 is considered significant). SigCleave analysis of DIA1R (data not shown) provided a highly significant score for cleavage between amino acids 31 and 32 (CSFS-LPAS, using single-letter amino-acid code, where the hyphen indicates the site of cleavage) of 10.23 (where values of greater than 3.5 are considered significant), suggesting this may represent the true site of SP cleavage for human DIA1R. Therefore, while all methods indicate amino-terminal SPs in both DIA1 and DIA1R, the exact SP cleavage sites will need to be determined experimentally. Together, our data indicate that both the *DIA1R* and *DIA1* gene products will be translocated into the ER, and transported to the Golgi apparatus.

### Ubiquitous tissue expression of human *DIA1* and *DIA1R*

To determine the cell types and tissues in which human *DIA1* and *DIA1R* are expressed, we explored the relevant expressed sequence tag (EST) profiles via UniGene [63] and searched two microarray databases: the GeneNote normal human expression database at the Weizmann Institute [75] and human Gene Atlas [76] at BioGPS [77].

EST profile data detected *DIA1* expression at levels of 61 transcripts per million (TPM) isolated from brain tissue (Table 2) and *DIA1R* at 25 TPM from brain tissue (Table 3). Of note, a wide analysis of human gene expression levels, in 14 different tissues, selected cut-off values for gene expression, with ‘low’ being ≤37 TPM and ‘high’ being ≥134 TPM [78]. Another human expression study indicated the majority (64%) of genes are expressed at between 3–50 TPM, 6% at levels between 100–5,000 TPM, and only 0.03% expressed at levels above 10,000 TPM [79]. Using these values as a guide, *DIA1R* can be described as being expressed at low-to-intermediate levels. By contrast, while *DIA1* is frequently expressed at low-to-intermediate levels, expression is high in mammary gland (135 TPM), ‘mouth’ (223 TPM; where libraries included in this tissue category are often derived from head or tongue), and vascular tissue (135 TPM). Ear expression of *DIA1* was the highest of those tissues falling in the intermediate range, and is close (123 TPM) to the ‘high expression’ category. These four tissue types, apparently expressing high levels of human *DIA1*, are all tissues with a high secretory output. One explanation is that Golgi apparatus-localized *DIA1* has a role in an aspect of secretion common to these tissues. Abnormalities in secreted levels of vascular growth factors and soluble vascular receptors have been reported in patients with ASD [80], as have abnormal levels of the endothelial cell protein PECAM-1 (platelet endothelial cell adhesion molecule-1) [81]. Defects in secretion or secreted factors are also considered major risk factors for otitis media [82], a condition occurring 2 to 3 times more frequently in those with ASD, than those without ASD [83]–[85]. Therefore, EST profiling results indicate widespread expression of human *DIA1* and *DIA1R* at moderate levels, including in brain tissue, with higher levels of *DIA1* in some tissues with a high secretory load.

**Table 2:** Expressed sequenced tag (EST) profiles showing *DIA1* gene expression in human tissues.

cDNA library source*	TPM**	EST/total***
adipose tissue	0	0/13105
adrenal gland	0	0/33195
ascites	0	0/40013
bladder	0	0/29757
blood	0	0/123476
bone	13	1/71655
bone marrow	81	4/48798
brain	59	66/1100969
cervix	41	2/48171
connective tissue	6	1/149254
ear	123	2/16212
embryonic tissue	23	5/215722
esophagus	0	0/20208
eye	37	8/211052
heart	33	3/89625
intestine	17	4/234477
kidney	56	12/211769
larynx	41	1/24144
liver	19	4/207739
lung	17	6/336969
lymph	0	0/44269
lymph node	54	5/91607
mammary gland	135	20/153267
mouth	223	15/67053
muscle	9	1/107711
nerve	0	0/15768
ovary	68	7/102050
pancreas	4	1/214811
parathyroid	48	1/20540
pharynx	0	0/41328
pituitary gland	0	0/16584
placenta	42	12/280828
prostate	21	4/189352
salivary gland	0	0/20155
skin	4	1/210574
spleen	0	0/53953
stomach	10	1/96622
testis	39	13/330449
thymus	36	3/81130
thyroid	0	0/81130
tonsil	0	0/16999
trachea	38	2/52412
umbilical cord	0	0/13680
uterus	12	3/232876
vascular	135	7/51779

*The EST profiles show approximate gene expression patterns, as inferred from EST counts from cDNA library sources, reported by sequence submitters to Unigene at the NCBI [63]. Libraries known to be normalized, subtracted, or otherwise biased are not included.

**TPM  =  transcripts per million.

***EST/total  =  number of *DIA1* ESTs in the total EST pool.

**Table 3:** EST profiles of *DIA1R* gene expression in human tissues.

cDNA library source*	TPM**	EST/total***
adipose tissue	76	1/13105
adrenal gland	0	0/33195
Ascites	0	0/40013
Bladder	0	0/29757
Blood	0	0/123476
Bone	0	0/71655
bone marrow	0	0/48798
Brain	25	28/1100969
Cervix	0	0/48171
connective tissue	40	6/149254
Ear	0	0/16212
embryonic tissue	0	0/215722
esophagus	0	0/20208
Eye	0	0/211052
Heart	78	7/89625
Intestine	8	2/234477
Kidney	9	2/211769
Larynx	0	0/24144
Liver	4	1/207739
Lung	5	2/336969
Lymph	0	0/44269
lymph node	0	0/91607
mammary gland	32	5/153267
Mouth	29	2/67053
Muscle	27	3/107711
Nerve	0	0/15768
Ovary	0	0/102050
pancreas	4	4/214811
parathyroid	0	0/20540
Pharynx	0	0/41328
pituitary gland	0	0/16584
Placenta	60	17/280828
Prostate	36	7/189352
salivary gland	0	0/20155
Skin	0	0/210574
Spleen	0	0/53953
Stomach	10	1/96622
Testis	0	0/330449
Thymus	0	0/81130
Thyroid	21	1/81130
Tonsil	0	0/16999
Trachea	19	1/52412
umbilical cord	73	1/13680
Uterus	17	4/232876
Vascular	57	3/51779

*The EST profiles show approximate gene expression patterns, as inferred from EST counts from cDNA library sources, reported by sequence submitters to Unigene at the NCBI [63]. Libraries known to be normalized, subtracted, or otherwise biased are not included.

**TPM  =  transcripts per million.

***EST/total  =  number of *DIA1* ESTs in the total EST pool.

**Figure 4:**
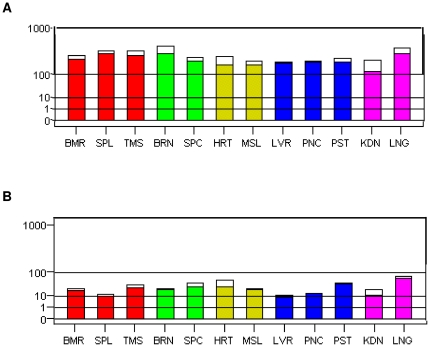
Microarray gene expression data for *DIA1* and *DIA1R*. Graphical presentation of microarray expression data for (A) *DIA1* or (B) *DIA1R* in normal, healthy human tissues. The intensity values (shown on the root-scale y-axis) are average values, where variation in the range of measurements is represented by a white box above the coloured minimal-measurement bar. Tissues are grouped according to their origin and the groups coloured: red  =  immune system; green  =  nervous system; yellow  =  muscle; blue  =  secretory glands; pink  =  ‘other’. Tissue abbreviation (on the x-axis) legend: BMR = bone marrow; SPL = spleen; TMS = thymus; BRN = brain; SPC = spinal cord; HRT = heart; MSL = skeletal muscle; LVR = liver; PNC = pancreas; PST = prostate; KDN = kidney; LNG = lung. Data were obtained from the GeneNote database of human genes at the Weizmann Institute [75], and are MAS5.0 normalized data obtained using the Affymetrix HG-U95 set A–E.

Microarray data from two sources (Figures 4, S2, and S3) supported the findings of the EST profiling results, as ubiquitous expression of both *DIA1* and *DIA1R* was found, including expression in brain tissue. Microarray expression levels of human *DIA1* were strikingly similar in all tissue types tested, but no confirmation of higher levels in ‘mouth’ tissues (tonsil, trachea, salivary gland, and tongue, being the comparable microarray tissue data), vascular tissue (CD105+ circulating endothelial cells being the closest microarray data), or mammary gland (no comparable microarray data) detected by EST profiling was found by microarray analysis. By microarray analysis, the highest level of *DIA1* was found in BDCA-4 (blood dendritic cell antigen 4 or neuropilin-1 or CD304) -positive plasmacytoid dendritic cells (Figure S2), which secrete high levels of type I interferons [86]. By contrast to the *DIA1* microarray data, microarray expression levels of *DIA1R* were less uniform (Figures 4 and S3). However, the highest *DIA1R* expression level found in the Gene Atlas microarray, which was found in liver (Figure S3), was not supported by the GeneNote microarray data (Figure 4). Within brain tissue, the highest expression level of *DIA1R* was in the cerebellar peduncles (Figure S3), a region of the brain abnormal in those with ASD [87]–[89]. However, microarray studies are plagued by a lack of reproducibility and accuracy [90]–[93], and we can not place too much emphasis on differences between gene expression levels, but must focus on overall expression profiles. While quantitative reverse-transcriptase polymerase chain reactions (RT-PCR) can provide more accurate and reproducible results [94], and can be used in conjunction with commercially available human tissue panels, analysis of data from this approach relies on the choice of reference gene for normalization [95]. Changes in the comparable housekeeping gene can lead to changes in the significance and expression of the target gene using RT-PCR approaches to quantifying gene expression [96], [97]. We can therefore only reliably conclude that human *DIA1* and *DIA1R* are both ubiquitously expressed genes.

### Expression of human *DIA1* and *DIA1R* during development

To examine expression of *DIA1* and *DIA1R* during human development, microarray data from the Gene Atlas [76] at the BioGPS [77] database (Figure S2 and S3) and EST profile data from UniGene [63], were examined (Tables 4 and 5). As EST data was restricted by the depth at which each developmental stage could be sampled, absences of expression cannot be assumed to indicate a lack of expression. Despite the limitations of EST profiling, the data revealed that both *DIA1* and *DIA1R* are expressed in fetal tissue, with *DIA1* expression also detected in the blastocyst (Tables 4 and 5). Microarray data confirmed expression of *DIA1* and *DIA1R* in fetal brain (Figure S2 and S3). Therefore, human *DIA1* and *DIA1R* are expressed in both adult and fetal tissues, including adult and fetal brain.

**Table 4:** EST profiles of *DIA1* gene expression during human development.

cDNA library source*	TPM**	EST/total***
embryoid body	42	3/70760
blastocyst	32	2/62318
fetus	44	25/564004
neonate	0	0/31097
infant	0	0/23620
juvenile	0	0/55555
adult	31	62/1939097

*The EST profiles show approximate gene expression patterns, as inferred from EST counts from cDNA library sources, reported by sequence submitters to Unigene at the NCBI [63]. Libraries known to be normalized, subtracted, or otherwise biased are not included.

**TPM  =  transcripts per million.

***EST/total  =  number of *DIA1* ESTs in the total EST pool.

**Table 5:** EST profiles of *DIA1R* gene expression during human development.

cDNA library source*	TPM**	EST/total***
embryoid body	0	0/70760
blastocyst	0	0/62318
Fetus	33	19/564004
Neonate	0	0/31097
Infant	0	0/23620
Juvenile	0	0/55555
Adult	23	45/1939097

*The EST profiles show approximate gene expression patterns, as inferred from EST counts from cDNA library sources, reported by sequence submitters to Unigene at the NCBI [42]. Libraries known to be normalized, subtracted, or otherwise biased are not included.

**TPM  =  transcripts per million.

***EST/total  =  number of *DIA1* ESTs in the total EST pool.

## Discussion

The *DIA1* gene, recently identified in a genetic study detecting autism genes by tracing recent shared ancestry, localizes to chromosome 3 at position 3q24 [61]. Using a bioinformatics approach, we have identified a *DIA1*-related gene, *DIA1R*, which localizes to the human X chromosome at position Xp11.3. As has been reported for mouse *DIA1* [64], we find both human *DIA1* and *DIA1R* are ubiquitously expressed, suggesting each gene has a generic cellular role. Furthermore, human *DIA1R*, like *DIA1* [62], encodes a signal peptide for targeting to the secretory pathway. Our comparison of human DIA1 and DIA1R at the amino acid level found conservation greatest between the central portions of the proteins, indicating this region may contribute to a biological role shared by each protein. The presence of *DIA1R* in the human genome may therefore explain why a homozygous deletion of a ubiquitously-expressed gene such as *DIA1* is not lethal [61]. However, human DIA1R, only has 62% similarity overall to human DIA1, and presumably cannot fully compensate for loss of *DIA1*, which would explain why *DIA1* deletion is associated with the neurological deficits of autism [61].

### Role of the *DIA1R* locus in autism and X-linked mental retardation

Around 10% of genes on the X chromosome cause XLMR if inactivated [60]. Human *DIA1R* is an X-linked gene at position Xp11.3, and is therefore part of the wider Xp11.3-Xp11.23 region. This wider region is implicated in a growing number of genetic diseases, including several eye disorders, XLMR, X-linked neuromuscular diseases, and susceptibility loci for schizophrenia and type 1 diabetes, with the Xp11.3 locus implicated specifically in XLMR [98], [99]. However, in addition to 19 characterized genes, there are at least four uncharacterized genes in this region of the X chromosome, including *DIA1R*, whose contribution to the disease phenotypes mapped to this region have not been assessed.

We next highlight a number of publications that support the hypothesis that deletion of the Xp11.3 region encompassing *DIA1R* causes ASD-like symptoms and/or XLMR. Essentially, we have carried out a retrospective genotype-phenotype analysis based on previously published studies which characterized the phenotypic traits of patients with deletions encompassing *DIA1R*. Genotype-phenotype mapping is a concept first introduced in 1991 [100], and genotype-phenotype correlation is widely used to examine the contribution of a certain mutation (genotype) to the resulting clinical traits (phenotype) [101]–[106]. Deletions encompassing a certain chromosomal region have variable phenotypes, depending on the size and position of the deleted region. However, features common to patients with deletions overlapping a locus of interest provide evidence for the specific effect of the loss of that gene or genes. The findings outlined below suggest *DIA1R* is joining an increasing number of genes able to cause both ASD and/or MR [45]–[48].

In the first study, examination of a patient with developmental delay, no expressive speech, mental retardation, and microcephaly, revealed a 4.0-Mb heterozygous deletion at Xp11.3–p11.4. This region included 5 candidate genes, including *DIA1R* and *CASK*, the latter of which was suggested to be causative due to its known role in synaptic function [107]. We suggest *DIA1R* deletion contributes to the phenotype of this patient.

Secondly, a linkage study of a four-generation family, implicated chromosomal region Xp11.3–11.21 in a novel neurological syndrome [108]. The syndrome is characterized by mild mental retardation, poor verbal skills, aggressive and/or agitated behaviour, and involuntary movements referred to as choreoathetosis. These symptoms overlap with those of ASD, and choreoathetosis is associated with Rett syndrome [109]. Reyniers and colleagues [108] discussed the possible role of 11 known genes with roles in neural signalling and neurotransmission, and how they might contribute to patient symptoms. The region does not include the *CASK* locus, but does include *DIA1R*, which can now also be considered a candidate gene for this novel syndrome.

A third study, by Zhang and colleagues [110], linked the region Xp11.3–Xq22.2 of male members of a large, cross-generational family group to poor verbal skills, mental retardation, and microcephaly. Zhang and coworkers discussed *OPHN1* as a candidate gene in this region, as it had previously been implicated in XLMR [110]. Nonetheless, the authors acknowledged that uncharacterized genes in this region may contribute to patient symptoms, and this may include *DIA1R*.

A fourth study further strengthens the argument that deletion of the Xp11.3 region, including *DIA1R*, is implicated in ASD-like symptoms and XLMR. This study identified a 5 Mb deletion at Xp11.3–11.4 in Turner syndrome (TS) patients [111], a region that does not involve *OPHN1*. TS is a relatively common genetic disorder, caused by X chromosome monosomy or other X chromosome defects. TS does not typically affect intellectual function or expressive verbal abilities, although it can be associated with reduced visual-spatial and executive skills, and demonstrable impairments in social skills [112]–[113]. Overall, TS is associated with a substantially increased risk of ASD [114]. Good and colleagues [111] found that deletion of Xp11.3–11.4 is associated with the development of ASD-like symptoms in TS patients, including poor eye contact, poor recognition of facial expression, and impaired social skills. Deletion of this region was also associated with increased amygdala volumes in TS patients, to levels typically associated with normal males [111]. Of note, many studies have also reported increased amygdala volumes in patients with ASD [115]–[118], but the phenomenon is not universal and the finding may be age-dependent [119].

Good and co-workers [111] hypothesized that there is a critical gene in the Xp11.3–11.4 region that is expressed in two copies in normal (46,XX) females, and that full dosage compensation does not occur in normal (46,XY) males or in Turner syndrome females (45,X), resulting in the increased amygdala volume found in normal males [120] and TS females [111]. They further suggested that this unknown gene has implications for autism and may contribute to the increased male susceptibility to ASD [111]. This gene cannot be *CASK*, as *CASK* undergoes X-inactivation [111]. As human *DIA1R* has recently been shown to escape X inactivation [121], *DIA1R* is a good candidate within this locus. Together, these findings demonstrate that *DIA1R* is a candidate gene for ASD-like symptoms and/or XLMR.

A more direct link between *DIA1R* and XLMR comes from two independent studies, where point mutations in *DIA1R* were implicated in XLMR [60], [122]. It should be noted that any ASD-like symptoms were not commented on in either of these studies, and may not have been evaluated or specifically ruled-out. In the first study, a mutation affecting the DIA1R signal peptide (a change of serine at position 24 to proline or ‘S24P’, using single amino acid abbreviations) was reported in a single XLMR patient, while the same mutation was absent in controls [122]. Our analyses reveal that the mutant *DIA1R* gene product (DIA1R-S24P) is still expected to have a functional signal peptide, but the mutation is predicted to change the signal peptide cleavage site (data not shown). Therefore, the DIA1R-S24P mutant is still expected to translocate to the secretory pathway, but the mutation may affect the size of the mature protein, with resulting impact on structure and function. In the absence of knowledge about ASD-like symptoms in this patient, the data currently suggest that mutation of *DIA1R* can cause XLMR, but complete deletion of *DIA1R* causes ASD-like symptoms with or without MR.

In the second study, a systematic large-scale screen for mutations implicated in XLMR found recurrent mutations in *DIA1R* [60]. In 67 XLMR patients a 383G>A mutation in *DIA1R* was found [60], where the mutation is part of an arginine-coding residue (codon: AGA), resulting in an arginine to lysine (mutated codon: AAA) change in the DIA1R gene product. The amino acid mutation (R128K) falls within the amino-terminal one-third of the DIA1R protein. While we are currently investigating the conservation of this amino acid in the wider DIA1 and DIA1R family, the findings of Tarpey and colleagues [60] suggest an R128K mutation alters DIA1R function in the human brain. These two studies demonstrate that independent *DIA1R* mutations are specifically associated with human XLMR, while the deletion studies implicate *DIA1R* deletion in autism-like syndromes and/or XLMR.

Overall, multiple lines of evidence now support a role for *DIA1R* in ASD and/or mental retardation: (i) the genotype-phenotype correlation data provided above; (ii) the high degree of amino acid conservation between DIA1 and DIA1R, where DIA1 is implicated in ASD [61]; (ii) targeting of both gene products to the Golgi apparatus, due to the conservation of signal peptide sequence in DIA1 and DIA1R; (iii) the ubiquitous tissue expression of both genes, including in brain tissue; (iv) and data from our accompanying paper which indicates an origin of *DIA1R* from a *DIA1* gene duplication early in the vertebrate lineage [123]. Each of these findings indicate conserved gene function. Therefore, it is not surprising that mutation of *DIA1R*, appears to cause a similar phenotype to deletion *DIA1*. (How these genes might function within the Golgi apparatus to cause this phenotype will be discussed below.)

### Disorders associated with the human 3q24 locus

In addition to the publication originally describing the homozygous deletion of *DIA1* in autism [61], wider deletions encompassing 3q24 (the region of human chromosome 3 encoding the *DIA1* gene), have also been reported. While each patient with a deletion encompassing 3q24 has unique features, presumably related to differences in deletion breakpoints between patients, several common features are found: developmental delay, mental retardation, and growth delay including microcephaly [124]–[126]. These three common symptoms overlap those described in a subset of PDD patients: females with Rett syndrome (where *MECP2* is mutated), patients with Angelman syndrome, and male Rett syndrome-like patients in which the *MECP2* gene is not implicated [127], [128].

By contrast, a patient with a deletion mapping cytogenetically to 3q25, showed developmental delay, but lacked the delayed growth and microcephaly of larger deletions in this chromosomal area [129]. Another patient with a deletion around 3q25 was specifically described as having autistic traits, severe learning difficulties, hypogonadism, and dysmorphic features [130]. These latter two deletions [129], [130] have only been characterized cytogenetically, which is a relatively imprecise method [131], and it would be beneficial to map the exact deletion boundaries to confirm whether the deleted regions encompass *DIA1*.

Therefore, while a single publication links homozygous *DIA1* deletion to autism [61], numerous studies report that deletion of portions of the 3q24 region encompassing *DIA1* are associated with developmental delay, ASD-like traits, and/or mental retardation [124]–[126]. Together, these publications provide further support for a role for *DIA1* in autism etiology, as first proposed by Morrow and colleagues in 2008 [61].

### Why does *DIA1* or *DIA1R* mutation cause autism-like syndromes?

We have demonstrated that human *DIA1* and *DIA1R* both encode signal peptides for targeting to the secretory pathway. Indeed, the *DIA1* gene product has been localized, using immunofluorescence microscopy, to the lumen of the Golgi apparatus [62]. Surprisingly, for genes with roles in neurological diseases, we find both *DIA1* and *DIA1R* are ubiquitously expressed, which indicates they fulfil basic, non-specialist cellular roles. However, despite ASDs being diagnosed on the behavioural manifestations of neurological deficits [132]–[135], many co-morbid non-neuronal conditions occur in ASD patients, suggesting widespread physiological and biochemical abnormalities in ASD patients [136]–[139]. Secretory pathway malfunction is a plausible explanation for the wide-ranging deficits in ASD and, as both DIA1 and DIA1R are predicted to play ubiquitous roles in some aspect of the cellular secretion pathway, they may be involved in a common cellular pathway deficient in those with ASDs.

The secretory pathway plays a key role in all cells, with essential roles in neuron development and function. Secretion in neurons, while having many factors in common to that in non-neuronal cells, has specific challenges. Recently, dendrites have been found to have satellite Golgi-like cisternal stacks, known as Golgi outposts, which have no membranous connection with the somatic Golgi [140]. What triggers Golgi outpost formation is not known, and only proteins common to the somatic Golgi have been identified in Golgi outposts [141]. Therefore, despite using the same secretory compartments as other cells, the dependence of neurons on the secretory pathway for synaptic transmission, outgrowth and remodelling of dendrites and axons, combined the vast distances involved, means the impact of mutation in many secretory pathway genes has a greater impact on neuronal, rather than non-neuronal, cell function [142]. Indeed, aberrations in cell secretion are being detected in increasing numbers of patients with ASD and/or mental retardation [143]–[149]. While large numbers of genes have been implicated as causative of ASD, we next discuss data suggesting that a common theme in ASD etiology may be alterations in cellular secretory pathway(s).

Recent work has found that four independent gene rearrangements, all previously implicated in ASD, induce the same morphological and functional aberrations in the large dense core granules of platelets [149]. It is also well-established that aberrant serotonin secretion from platelets is common in those with ASD [150]–[151], along with aberrant levels of other secreted factors, including neuropeptides and neurotrophins [137], [143], [144]. Furthermore, two research groups have found evidence for defective enzymatic activity in the secretory pathway of those with autism. The more recent of these two studies, found a functional deficit in a secretory pathway-localized kinase by assaying substrates in the saliva of patients with ASD [152]. The activity of this ubiquitously expressed kinase can affect the traffic and/or function of its substrates [153]–[157]. The second research group found a deficit in the activity of tyrosylprotein sulfotransferase (TPST) in platelets of patients with ASD [158], [159]. TPST is a ubiquitious sulfotransferase, which acts on substrates in the lumen of the Golgi apparatus [160]. Protein tyrosine sulfation is again known to affect the function of the target protein [161]. Therefore, an emerging theme in autism is defects in the delivery or function of secreted, or cell-surface, molecules. The location of DIA1 and DIA1R suggests they may also affect such processes.

Further evidence supporting a role of secretory pathway deficits in ASD etiology, are studies identifying genes related to glycobiology in autism [48], [162]. As the Golgi apparatus is the main site of glycosylation [163], this finding further implicates deficits in Golgi apparatus function in ASD etiology. Therefore, a unifying hypothesis is that genes involved in the pathogenesis of autism are involved in regulation of secretion, the expression of secreted proteins, or the optimal function of proteins trafficking via the secretory pathway. We propose a model where DIA1 and DIA1R play a role in regulation of cargo secretion or modification of cargo molecules, which is required for optimal cargo functionality. We conclude that defects in *DIA1* or *DIA1R* function within the lumen of the Golgi apparatus of human cells causes autism and/or mental retardation. However, in the absence of known protein motifs and domains (data not shown), ongoing studies are required to determine the exact cellular roles of human *DIA1* and *DIA1R*, particularly the effects of mutation on brain function.

## Materials and Methods

### Detection and chromosome mapping of human *DIA1R*

The human DIA1 amino acid sequence [61], [62] was used in a BLAST search, using the BLASTP [65] program, and this identified the related human sequence, DIA1R, on the non-redundant protein database at the NCBI [63]. Chromosomal location and chromosome ideograms were obtained using MapViewer at NCBI [63].

### Alignments, protein analysis, and homologue identification

Protein sequence alignments were generated with CLUSTALX version 1.8 [164]. The ExPASy Compute pI/MW tool [165] was used to calculate theoretical molecular weights. Three trans-membrane prediction methods were used to analyze protein sequences: TMpred [166], TMAP [167], and HMMTOP version 2.0 [168]. Signal peptides were evaluated using SignalP version 3.0 [72] or the SigCleave algorithm [73] which is part of the EMBOSS software suite [74]. Amino acid motifs and domains were investigated using the following resources: MOTIF at GenomeNet [169]; PSORT-II [170]; the Conserved Domain Database at the NCBI, which also contains data from Pfam, SMART and COG [171]; and the Eukaryotic Linear Motif resource [172]. Homologs of human *DIA1* and *DIA1R* were identified using the NCBI HomoloGene resource [63].

### Human tissue expression

EST profiles were obtained via the UniGene database at NCBI [63] and were accessed on 12^th^ November 2010. Microarray data was accessed from the GeneNote normal human expression database at the Weizman Institute [75] and the Gene Atlas database [76] via the BioGPS gene portal server [7].

## Supporting Information

Figure S1 Neural network prediction of signal peptides in human DIA1 and DIA1R. The amino acid sequence of (A) human DIA1 or (B) human DIA1R, was evaluated for amino-terminal signal peptides using the neural network (NN) algorithm of SignalP v3.0 [72]. The C-score (cleavage score for each amino acid) is indicated in red, the S-score (signal peptide score) is indicated in green, and the Y-score (derived from the C-score and S-score, and can give a better indication of the cleavage site) is indicated in blue. The significance cut-off value for all probability scores is 0.5, and is indicated by a pink dotted line. Standard single-letter amino acid abbreviations and numbering are provided below the graph.
(0.02 MB PDF)

Figure S2 BioGPS microarray expression data for *DIA1*. Graphical presentation of expression data for *DIA1* in a variety of normal, and cancerous human tissues and cells. Expression values were obtained from an Affymetrix U133A microarray and relate to fluorescence intensity. Multiple probes were used for each transcript on the microarray and these intensity values have been normalized, background-subtracted, and summarized using the data-processing algorithm GCRMA (GeneChip Robust Multi-array Average). The identifier of the Affymetrix probe set used is indicated above the graph. Data were obtained from the Gene Atlas database of human genes [76] at the BioGPS gene portal server [77].
(0.02 MB PDF)

Figure S3 BioGPS microarray expression data for *DIA1R*. Graphical presentation of expression data for *DIA1R* in a variety of normal, and cancerous human tissues and cells. Expression values were obtained from an Affymetrix U133A microarray and relate to fluorescence intensity. Multiple probes were used for each transcript on the microarray and these intensity values have been normalized, background-subtracted, and summarized using the GCRMA (GeneChip Robust Multi-array Average) data-processing algorithm. The identifier of the Affymetrix probe set used is indicated above the graph. Data were obtained from the Gene Atlas database of human genes [76] at the BioGPS gene portal server [77].
(0.03 MB PDF)
