# Supplementary material for: DIA1R Is an X-Linked Gene Related to Deleted In Autism-1
Source: PLoS One. 2011 Jan 17;6(1):e14534. doi: 10.1371/journal.pone.0014534 (PMC3022024; doi:10.1371/journal.pone.0014534)
Supplement: Figure S1 — Neural network prediction of signal peptides in human DIA1 and DIA1R. The amino acid sequence of (A) human DIA1 or (B) human DIA1R, was evaluated for amino-terminal signal peptides using the neural network (NN) algorithm of SignalP v3.0 [72]. The C-score (cleavage score for each amino acid) is indicated in red, the S-score (signal peptide score) is indicated in green, and the Y-score (derived from the C-score and S-score, and can give a better indication of the cleavage site) is indicated in blue. The significance cut-off value for all probability scores is 0.5, and is indicated by a pink dotted line. Standard single-letter amino acid abbreviations and numbering are provided below the graph. (0.02 MB PDF) [file pone.0014534.s001.pdf]

Figure S1. Neural network prediction of signal peptides in human DIA1 and DIA1R. The amino acid sequence of (A) human DIA1 or (B) human DIA1R, was evaluated for amino-terminal signal peptides using the neural network (NN) algorithm of SignalP v3.0 [51]. The C-score (cleavage score for each amino acid) is indicated in red, the S-score (signal peptide score) is indicated in green, and the Y-score (derived from the C-score and S-score, and can give a better indication of the cleavage site) is indicated in blue. The significance cut-off value for all probability scores is 0.5, and is indicated by a pink dotted line. Standard single-letter amino acid abbreviations and numbering is provided are provided below the graph.

Fig. S1

A

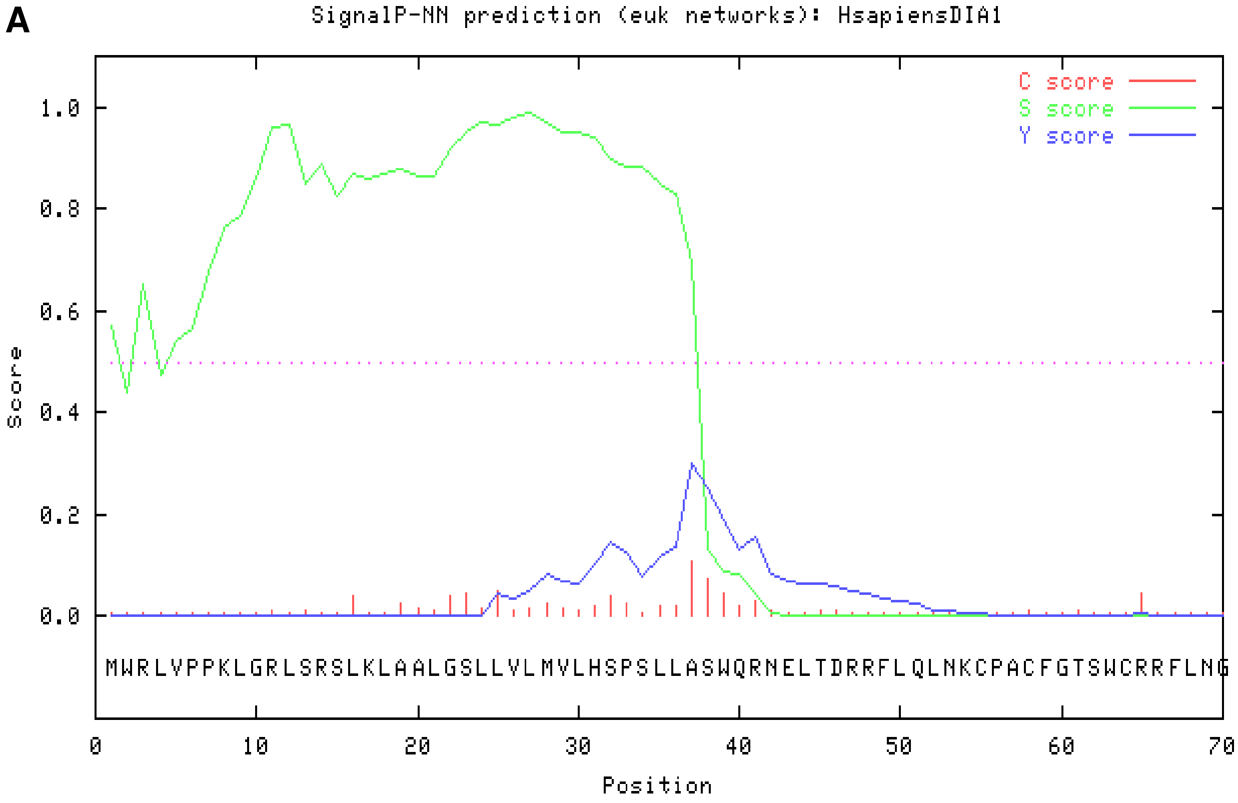
SignalP-NN prediction (euk networks): HsapiensDIA1
C score
S score
Y score
Score
1.0
0.8
0.6
0.4
0.2
0.0
MWRLVPPKLGRLSRSLKLAALGSLLVLMVLHSPSLLASWQRNELTDRRFLQLNKCPACFGTSWCRRFLNG
0
10
20
30
40
50
60
70
Position

B

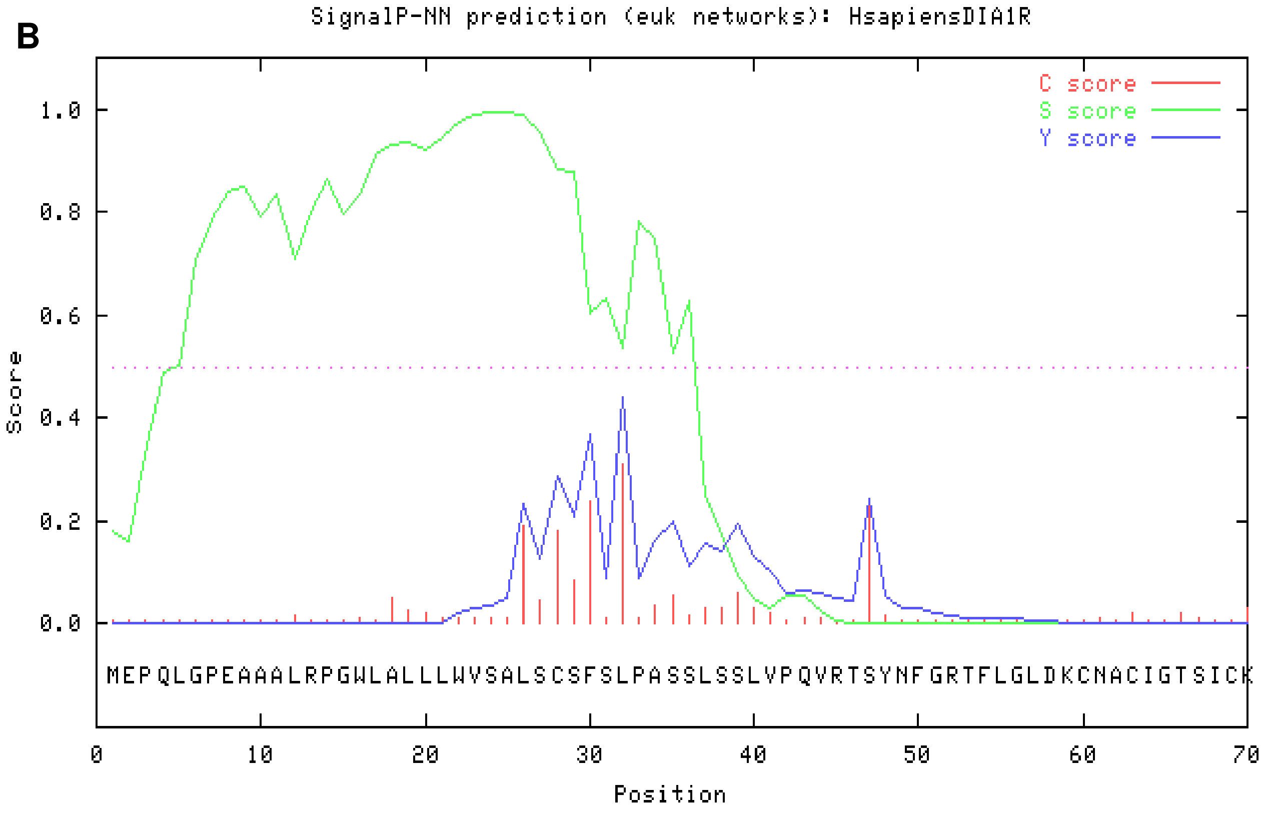
SignalP-NN prediction (euk networks): HsapiensDIA1R
C score
S score
Y score
Score
1.0
0.8
0.6
0.4
0.2
0.0
MEPQLGPEAAALRPGWLALLLWVSALSCSFSLPASSLSSLVPQVRTSYNFGRTFLGLDKCNACIGTSICK
0
10
20
30
40
50
60
70
Position
