# Supplementary material for: DIA1R Is an X-Linked Gene Related to Deleted In Autism-1
Source: PLoS One. 2011 Jan 17;6(1):e14534. doi: 10.1371/journal.pone.0014534 (PMC3022024; doi:10.1371/journal.pone.0014534)
Supplement: Figure S2 — BioGPS microarray expression data for DIA1. Graphical presentation of expression data for DIA1 in a variety of normal, and cancerous human tissues and cells. Expression values were obtained from an Affymetrix U133A microarray and relate to fluorescence intensity. Multiple probes were used for each transcript on the microarray and these intensity values have been normalized, background-subtracted, and summarized using the data-processing algorithm GCRMA (GeneChip Robust Multi-array Average). The identifier of the Affymetrix probe set used is indicated above the graph. Data were obtained from the Gene Atlas database of human genes [76] at the BioGPS gene portal server [77]. (0.02 MB PDF) [file pone.0014534.s002.pdf]

Figure S2. BioGPS microarray expression data for *DIA1*. Graphical presentation of expression data for *DIA1* in a variety of normal, and cancerous human tissues and cells. Expression values were obtained from an Affymetrix U133A microarray and relate to fluorescence intensity. Multiple probes were used for each transcript on the microarray and these intensity values have been normalized, background-subtracted, and summarized using the data-processing algorithm GCRMA (GeneChip Robust Multi-array Average). The identifier of the Affymetrix probe set used is indicated above the graph. Data were obtained from the Gene Atlas database of human genes [55] at the BioGPS gene portal server [56].

Fig. S2

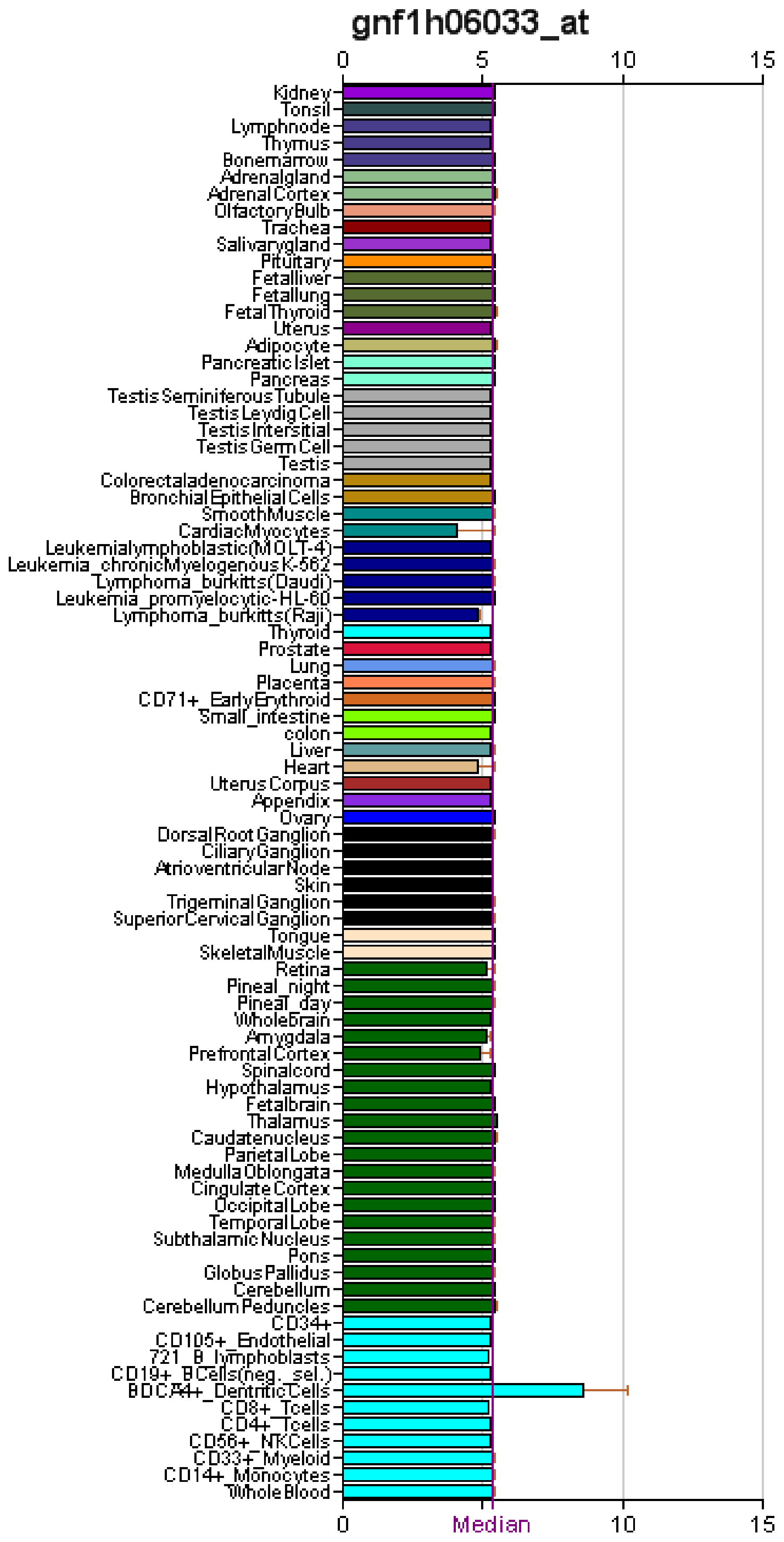
gnf1h06033_at
0
5
10
15
Kidney
Tonsil
Lymphnode
Thymus
Bonemarrow
Adrenalgland
AdrenalCortex
OlfactoryBulb
Trachea
Salivarygland
Pituitary
Fetalliver
Fetallung
FetalThyroid
Uterus
Adipocyte
PancreaticIslet
Pancreas
TestisSeminiferousTubule
TestisLeydigCell
TestisIntersitial
TestisGermCell
Testis
Colorectaladenocarcinoma
BronchialEpithelialCells
SmoothMuscle
CardiacMyocytes
Leukemialymphoblastic(MOLT-4)
Leukemia_chronicMyelogenousK-562
Lymphoma_burkitts(Daudi)
Leukemia_promyelocytic-HL-60
Lymphoma_burkitts(Raji)
Thyroid
Prostate
Lung
Placenta
CD71+_EarlyErythroid
Small_intestine
colon
Liver
Heart
UterusCorpus
Appendix
Ovary
DorsalRootGanglion
CiliaryGanglion
AtrioventricularNode
Skin
TrigeminalGanglion
SuperiorCervicalGanglion
Tongue
SkeletalMuscle
Retina
Pineal_night
Pineal_day
WholeBrain
Amygdala
PrefrontalCortex
Spinalcord
Hypothalamus
Fetalbrain
Thalamus
Caudatenucleus
ParietalLobe
MedullaOblongata
CingulateCortex
OccipitalLobe
TemporalLobe
SubthalamicNucleus
Pons
GlobusPallidus
Cerebellum
CerebellumPeduncles
CD34+
CD105+_Endothelial
721_B_lymphoblasts
CD19+_BCells(neg._sel.)
BDCA4+_DentriticCells
CD8+_Tcells
CD4+_Tcells
CD56+_NKCells
CD33+_Myeloid
CD14+_Monocytes
WholeBlood
0
Median
10
15
